# Effects of Adding Micronutrient Mixtures to a Model Dark Chocolate System and Partially Replacing the Fat Phase with a Structuring Oleogel

**DOI:** 10.3390/foods14030430

**Published:** 2025-01-28

**Authors:** Paulo Henrique Silva Santos, Cristina Kaori Suzuki, Suzana Caetano da Silva Lannes

**Affiliations:** Pharmaceutical Sciences School, University of Sao Paulo, Av. Prof. Lineu Prestes, 580 Butantã, São Paulo CEP 05508000, Brazil; paulo_santos@usp.br (P.H.S.S.); cristinasuzuki@usp.br (C.K.S.)

**Keywords:** chocolate, oleogel, rheology, structure, thermal analysis, nutritional improvement

## Abstract

Supplements improve consumers’ health and well-being. Oleogels are fat substitutes that offer nutritional and structural improvements to foods. This study aimed to formulate and observe chocolate’s structural differences and properties supplemented with different premixes for immune support and oleogel based on Brazil nut oil. Six 60% dark chocolates were produced using oleogel as a partial substitute for cocoa butter (with and without premixes), and premix 1 (vitamin D3, vitamin C, and zinc) or premix 2 (vitamins D3, C, A, E, zinc, and selenium). Texture, rheology, thermal analysis DSC, color, water activity, moisture, pH, and fat profile were determined. The results revealed that the whiteness index was higher for the oleogel and supplemented products. The use of oleogel reduced the lipid content of the products by 5% and saturated fatty acids by 13%. DSC showed changes in the melting and crystallization profiles for the supplemented products. All samples showed thixotropy, and the yield value was significantly different (*p* ≤ 0.05) in only one sample. Hardness presented a lower value (±50%) for products with oleogel. In sum, replacing part of the cocoa butter with an oleogel made the products softer, improved their structural quality, and changed their melting and crystallization profiles, and the chocolates showed nutritional improvement.

## 1. Introduction

Chocolate is a universally appreciated food, whose popularity exceeds cultural and geographical boundaries. Combining cocoa solids, sugar, and milk dispersed in a continuous lipid phase, its unique composition results in an unparalleled sensory experience, making it a true gastronomic delight [1,2]. However, as global concerns about health and well-being grow, the food industry’s challenge is finding ways to improve the nutritional quality of foods without compromising their palatability [3].

Studies have shown that food fortification is an effective strategy to combat nutritional deficiencies [4]. Adding micronutrients to foods, such as cereals and milk, has successfully reduced the prevalence of deficiencies, especially in vulnerable populations such as children and pregnant women. Several studies indicate that the consumption of chocolate, particularly dark chocolate, is associated with lower blood pressure and a reduced risk of cardiovascular disease and type 2 diabetes [5,6,7,8]. In Brazil, due to the substitution of cocoa butter with vegetable fats in chocolate formulations, the product loses nutrients during the process that can be added back later [7].

The discovery of cocoa and chocolate contributed to changing the way humans relate to food. Among the unique factors that distinguish them are their sensory, nutritional, and functional profiles and the presence of flavonoids such as epicatechin, catechin, and procyanidins, which are antioxidant compounds found in cocoa pulp and affect vascularization [6].

The addition of bioactive compounds to food formulations has been the subject of research by scientists worldwide, aiming to provide consumers with healthy and practical alternatives in their daily lives [5,7,8]. Presently, the Brazilian economic crisis scenario has led to a change in consumer profiles, making them more conscious and rational. Thus, developing a product as beloved as chocolate that offers nutritional benefits without losing its sensory characteristics has become a technological challenge. Furthermore, the search for alternatives to reduce saturated fat content without compromising the structure and texture of chocolate has led to the development of new ingredients, such as structuring oleogels. These are crystalline lipid systems capable of providing stability and desirable texture to foods without compromising flavor, sensory quality, and microstructure. Lipid is the continuous phase of oil-based oleogels and exhibits the physical properties of hydrogels that have a continuous liquid aqueous phase and are based on rigid networks of crystalline triacylglycerol. It can be used in food formulations supporting texture, spreadability, crunchiness, shelf life, and product flavor. Structuring fat foods (ice cream, fillings, and spreads) is based on crystalline networks of high-melting-point triacylglycerols, which should be part of the oleogels [9,10,11,12].

Researchers have emphasized the benefits of structuring oleogels for the food industry [13,14,15]. These systems have shown potential as substitutes for saturated fat in various food products, offering texture and calorie reduction improvements, thereby ensuring healthier options for consumers [16].

Rheology has been used to study the structure of foods. Some procedures can be chosen, and thixotropy, which linearly ramps the shear rate in a period interval, upwards and downwards, determines the resulting up- and down-shear stress curves. The up-curve, above the down-curve, defines a hysteresis area proportional to the energy required to break down the thixotropic structure. The two determinant parameters, viscosity and yield value, in the chocolate system can be obtained by a Casson equation [11,12]. Some methods can be used to measure rheology in chocolate [17].

Thixotropy is a phenomenon that is sometimes necessary for industry and at other times undesirable. In a material at rest, when the flow starts, the viscosity begins to decrease with time, and the recovery occurs when the flow is discontinued. When the shear is relaxed, there is a breakdown of the internal structure of the fluid or a rearrangement of the structure from smaller units. A thixotropic liquid is defined by its potential to have a reversible gel structure whenever the substance is left at rest for a long time. The change from gel to sol state and from sol to gel is reproducible. Both the breakdown and recovery processes are slow relative to the rate of change of stress, or the observation time at constant stress [17,18,19].

Therefore, this study aimed to investigate the effects of adding micronutrient blends to a model chocolate system and partially replacing the lipid phase with a structuring oleogel. The goal was to formulate and observe the structural differences and properties of chocolate supplemented with different premixes for immune support in an oleogel based on Brazil nut oil. The focus was on improving the chocolate’s nutritional, physical, and structural properties.

## 2. Materials and Methods

### 2.1. Oleogel Preparation

The oleogel was prepared following the procedure described by Espert, Salvador, and Sanz [20], with some process adaptations. The initial emulsion was prepared with 47% (*w*/*w*) Brazil nut oil (Federal University of Para, Belém-PA, Brazil), 1.5% (*w*/*w*) HPMC (hydroxypropyl methylcellulose) (Metachem, Itupeva-SP, Brazil), and water to reach 100% (*w*/*w*). For the initial emulsion formation, HPMC was gradually dispersed into the Brazil nut oil under continuous agitation using a mechanical stirrer (Fisaton, São Paulo-SP, Brazil) at a speed of 280 rpm for 5 min. Subsequently, water at 10 °C was added for proper cellulose hydration, and the mixture was homogenized with an Ultra-Turrax (Marconi, Piracicaba-SP, Brazil), using the S18N-19G dispersion tool, until a white and viscous emulsion was obtained (6500 rpm for 1 min, followed by 3 min at 17,500 rpm). The emulsion was then placed in a 40 cm × 20 cm aluminum mold and dried in an adiabatic dryer at 60 °C until the moisture content was reduced to below 5%, which took approximately 48 h.

### 2.2. Chocolate Preparation

Six formulations containing 60% cocoa solids were produced for the initial study of the chocolates, with variations in cocoa butter content when partially replacing it for oleogel, and the addition of vitamin and mineral powder premixes. Table 1 outlines the percentages of the components used in the formulations and the ingredients involved in their production. The ingredients included cocoa butter (IBC—Indústria Brasileira de Cacau, Rio das Pedras-SP, Brazil), cocoa liquor (IBC—Indústria Brasileira de Cacau, Rio das Pedras-SP, Brazil), soy lecithin (Barentz, São Paulo-SP, Brazil), PGPR (polyglycerol polyricinoleate) (Barentz, São Paulo-SP, Brazil), powdered vanillin flavor (Mix Aromas, São Bernardo do Campo-SP, Brazil), refined sugar (Camil Alimentos, São Paulo-SP, Brazil), premix 1 for immunity (vitamins D3 and C, and zinc) (VITAQUIMA/DSM, Brazil), and premix 2 for immunity (vitamins C, D3, A, and E, selenium, and zinc) (VITAQUIMA/DSM, São Paulo-SP, Brazil).

The amounts of cocoa butter used for the samples were calculated to ensure that the final product would have a lipid content close to 35%. This approach resulted in a Control dark chocolate with 60% cocoa content.

### 2.3. Chocolate Production

The Food Technology Laboratory III, located at the Faculty of Pharmaceutical Sciences, University of São Paulo, Brazil, sources raw materials from reliable suppliers for chocolate production. The laboratory operates with a more streamlined manufacturing process, requiring less space, time, and money. The chocolates are formulated using the universal mixer with a ball mill, model WA-FA20 (Mazzetti, Milan, Italy). During production, the formulation ingredients were sequentially added to the equipment, with a working temperature of 45 °C. For T1, T2, T3, and T4, the powder premixes were added after the chocolate was removed from the equipment, and were mixed in a planetary mixer (Erly, São Paulo, Brazil). Chocolate tempering was performed manually on a marble slab. Following pre-crystallization, the chocolate was molded and cooled at 5 °C ± 3 °C for 20 min, after which it was demolded and packaged. All chemical and physical analyses of both the chocolate and the fats were performed in triplicate.

Illustration 1 shows the sequency of chocolate manufacture.

Illustration 1—Sequency of chocolate production (WA-FA20). 
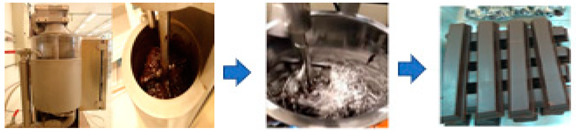

### 2.4. Nutritional Composition (Ash and Moisture)

The physicochemical analyses for determining the nutritional composition (ash and moisture) of the dark chocolate were carried out following the methodologies outlined by AOAC (2005) [21].

### 2.5. Total Lipids and Fatty Acid Profile

Total lipids from the dark chocolate were extracted using the 36 C/04 method, as described by Schetty et al. [22] and adapted by Lannes [23].

The analysis was conducted on samples C (Control) and T3 (chocolate with 3% oleogel). Sample C (Control) was chosen because it has the same lipid content as samples T1 and T2, which contain vitamin and mineral premixes but no oleogel addition. This sample served as a baseline to evaluate the lipid properties of samples with premix additions, without the interference of oleogel, allowing for a more direct comparison of the impact of the different premixes on the lipid profile. Sample T3 (chocolate with 3% oleogel) was selected because it has the same lipid content as sample C, but with the addition of oleogel. The choice of this sample was due to the fact that oleogel was used as a partial substitute for cocoa butter, which could modify the physical structure of the chocolate and, consequently, its lipid profile. Analyzing sample T3 allowed us to observe the changes in lipid content and nutritional properties due to the presence of oleogel, without altering the total fat content compared to the Control. Therefore, C and T3 were selected for lipid analysis because both had the same lipid content, allowing for a more accurate evaluation of the effects of oleogel addition on the lipid profile of the chocolate. The comparison between these samples enabled a detailed analysis of the changes caused by the partial replacement of cocoa butter with oleogel, while keeping other factors, such as lipid content, constant.

The extraction process began with the treatment of 10 g of ground chocolate samples with 75 mL of concentrated HCl (37%) and 200 mL of distilled water. The mixture was boiled for 20 min and then filtered using 3 L of boiling water through a 24 cm diameter filter. After filtration, the samples were dried in an oven at 75 °C for 12 h. Lipid extraction was performed using petroleum ether for approximately 4 h in a Soxhlet apparatus. After solvent evaporation, the residue was kept in an oven at 100 °C for 1 h until it reached a constant weight. The final weight was recorded after cooling in a desiccator.

The fatty acids were methoxylated, or converted into methyl esters, according to the methodology of Hartman and Lago [24], using 0.5N KOH and a reagent solution in methanol. The analyses were performed on a Varian gas chromatograph, model 430 GC, equipped with an automatic injector, a flame ionization detector, and “Varian’s Galaxie Chromatography Software.” A fused silica capillary column SP-2560 (Supelco, Darmstadt, Germany) was used, measuring 100 cm in length × 0.25 mm internal diameter, with 0.2 µm polyethylene glycol within the column. The conditions were as follows: split injection with a 50:1 ratio; column temperature: 140 °C for 5 min, programmed to increase to 240 °C at a rate of 4 °C/min; carrier gas: helium, at an isobaric pressure of 37 psi; linear velocity of 20 cm/s; makeup gas: helium at 29 mL/min; injector temperature: 250 °C; and detector temperature: 250 °C. The qualitative composition was determined by comparing the retention times of peaks with the respective fatty acid standards. The quantitative composition was calculated by area normalization, expressed as a mass percentage, following the official method AOCS Ce 1–62 [25].

### 2.6. Triacylglycerol Determination

Triacylglycerol was determined using the LAMES software platform (Federal University of Goiás, Goiânia, Brazil), with statistical analysis to evaluate the lipid profiles. The use of this tool aimed to identify the specific characteristics of the triacylglycerols present in the samples, considering the changes in the physical and lipid properties of chocolate with the addition of oleogel and premixes. The analysis was conducted to compare the variations in the C (Control) and T3 (with 3% oleogel) samples to assess the lipid structure and its nutritional implications.

### 2.7. Water Activity Determination

Water activity was measured using a Novasina LabMaster device (Novasina, Switzerland) at 25 °C.

### 2.8. Color Analysis

The color spectrum was determined using a Colorquest XE device with 0/45° geometry, and the data were analyzed using Universal Software V4.10 (Hunter Lab, Reston, VA, USA). The whiteness index (WI) was calculated using Equation (1), and the color difference (ΔE) was calculated using Equation (2). From the values of a* and b*, the chroma (C*) and hue angle (h°) coordinates were obtained using Equations (3) and (4).

Equation (1)—whiteness index

(1) $$WI=100-[(100-L^{*\;}{)}^{2}+a{*}^{2}+b{*}^{2\;\;\;}]$$

Equation (2)—color difference

(2) $$∆E+\sqrt{\left(L^{1}+L^{2}\right)+\left(a^{1}+a^{2}\right)+\left(b^{1}+b^{2}\right)}$$

Equation (3)—coordinate chroma

(3) $$d*=\sqrt{(a^{*}{)}^{2}+(b^{*}{)}^{2}}$$

Equation (4)—hue angle

(4) $$h{}^{\circ}={tan}^{-1}\frac{\left(b^{*}\right)}{\left(a^{*}\right)}$$

### 2.9. Thermal Analysis (DSC—Differential Scanning Calorimetry)

Thermal analyses to determine the melting and crystallization points of the chocolate were conducted using a device DSCi Series—Instrument Specialists Incorporated, I Series (Twin Lakes, WI, USA). After calibrating the equipment at a scanning rate of 5 °C/min using an aluminum pan as a reference, approximately 5 mg of the samples was loaded into 40 µL aluminum pans, sealed with lids, and heated at a rate of 20 °C/min from 25 °C to 60 °C. The samples were maintained at 60 °C for 10 min, then cooled from 60 °C to −10 °C at a rate of 10 °C/min, held at −10 °C for 30 min, and subsequently reheated from −10 °C to 60 °C at a rate of 20 °C/min, all under a nitrogen atmosphere (N2). Onset, peak, and end temperatures were calculated automatically by Thermal Analysis Infinity PRO Software for Windows Version 4.2.213 (Instrument Specialists Inc., Twin Lakes, WI, USA).

### 2.10. Physical and Structural Analyses of Chocolates

#### 2.10.1. Texture Determination

Fracture tests on chocolate bars were conducted using a TA-XT2 texture analyzer (Stable Micro Systems, Godalming, UK) with an HDP/3PB probe, and the samples were conditioned at 25 °C. The test parameters were as follows: pre-test, test, and post-test speeds: 2.0 mm/s; distance: 10 mm; load cell: 25 kg; trigger force: 0.05 N; and compression force—return to start. Data were collected using the “Texture Expert Exceed” software, version 2.64 (Stable Micro Systems, Godalming, UK). All analyses were performed on tempered chocolate bars measuring 10.0 cm × 1.2 cm × 0.9 cm [26].

#### 2.10.2. Rheological Analysis

Rheological tests were conducted using a Haake-MARS II Rheometer with RheoWin Job Manager software (ThermoScientific, Karlsruhe, Germany), with a thermostated bath set at 40 °C. The analysis used the Casson equation to fix the points obtained. The test employed a cone-plate sensor (C35/1 Ti polished), with a gap of 0.024 mm, and a controlled rate (CR) rotational test in three steps: (1) 0.00 1/s to 65.00 1/s; t = 180 s; (2) 65.00 1/s; t = 60 s; and (3) 65.00 1/s to 0.00 1/s; t = 180 s. The sample was placed on the fixed plate of the equipment, in sufficient quantity to cover an area of approximately 2 cm² and fill the gap between the plates [27].

Equation (5)—Casson Equation

(5) $${Ʈ}^{0.5}=\;{{\tau_{o}}^{0.5}+{ƞ}_{c}\;}^{0.5}\;\;\Upsilon^{0.5}$$

Ʈ = Shear stress;
Ʈo = Yield stress;
Ƞc = Plastic viscosity;
Υ = Shear rate.

### 2.11. Determination of Zinc and Selenium

The determination of zinc and selenium was performed using the ICP-OES technique, which is based on the multi-elemental analysis of the sample composition through the emission of excited atoms and ions. Sample digestion was carried out using concentrated nitric acid and 30% hydrogen peroxide under heating at 200 °C in a microwave oven with closed vessels. Experiments were conducted in duplicate for each sample, with a mass of 200 mg diluted to 50 mL. The analysis was performed using an Inductively Coupled Plasma Optical Emission Spectrometer (ICP OES, Radial) from Spectro, model Arcos (AMETEK, Berwyn, PA, USA). The detection limit (LD: 0.01 mg/L), the quantification limit (LQ: 0.10 mg/L), and the results were expressed in parts per million (ppm); ppm = mg/L or mg/kg; 1 ppm = 0.0001.

### 2.12. Statistical Analysis

All analyses were performed in triplicate, calculating means and standard deviations. The results were analyzed using ANOVA and, subsequently, Tukey’s test, with *p* ≤ 0.5, using Minitab 17 software (Minitab Inc.,State College, PA, USA).

## 3. Results

### 3.1. Moisture, Water Activity, and pH Analysis

Physicochemical results are important to verify the behavior of the sample in the face of changes in formulation. Table 2 presents the results of moisture, water activity (Aw), and pH determinations of the samples.

### 3.2. Color Analysis

The results of the colorimetric analysis of chocolates that underwent various modifications are presented in Table 3.

The hue angle (h°) was defined as starting at the +a axis and is expressed in degrees, where 0° corresponds to +a (red), 90° corresponds to +b (yellow), 180° corresponds to −a (green), and 270° corresponds to −b (blue).

### 3.3. Thermal Analysis DSC

#### 3.3.1. Melting Analysis

Table 4 shows the melting thermal analysis results—Differential Scanning Calorimetry—of the samples.

#### 3.3.2. Crystallization Thermal Analysis

Table 5 presents the results of the crystallization thermal analysis—Differential Scanning Calorimetry—of the samples.

### 3.4. Rheological Analysis

**Figure 1:**
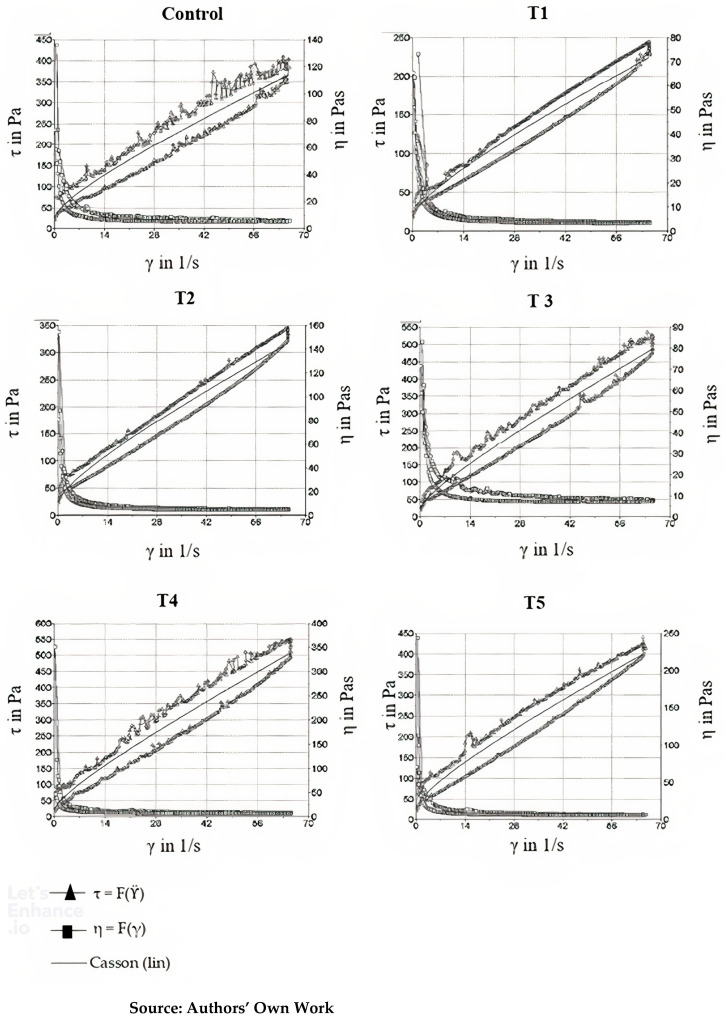
Thixograms and Casson curves of samples.

Table 6 shows the results of the rheological analysis of the samples, with Casson viscosity, Casson yield stress, and thixotropy. Figure 1 presents the thixograms obtained in the thixotropy analysis of the samples.

### 3.5. Texture

**Figure 2:**
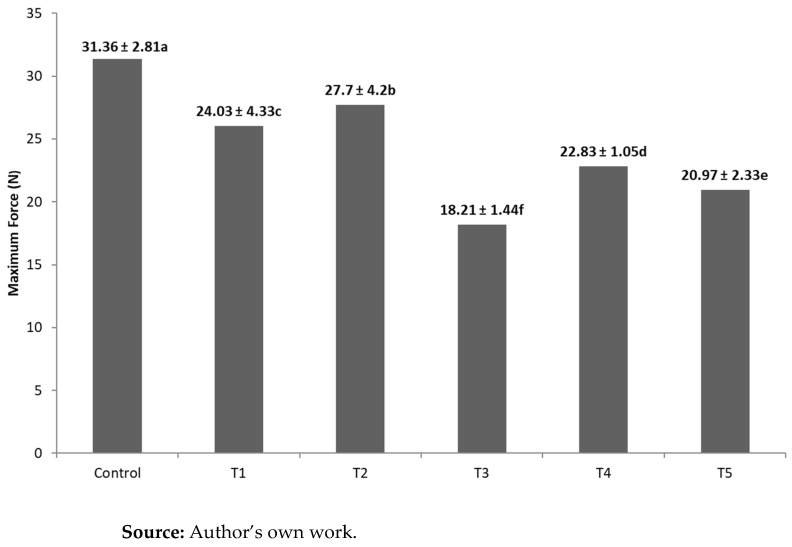
Hardness of samples. a–f indicate values statistically significant differences (*p* ≤ 0.05).

In the texture analysis, a maximum force (N) to break the samples was obtained, pointing to the hardness of the chocolates (Figure 2).

### 3.6. Total Lipids, Fatty Acid Profile, and Triacylglycerols

Table 7 and Table 8 clearly show the fatty acid profile of the Control and T3 samples, as well as the triacylglycerol composition of the same samples. The T3 sample was selected because cocoa butter was partially replaced by oleogel, which led to changes in its lipid profile.

### 3.7. Determinations of Selenium and Zinc

Minerals were added to the samples to complement the daily intake requirement per person since chocolate is consumed in small portions. Table 9 shows the results obtained.

## 4. Discussion

### 4.1. Moisture, Water Activity, and pH Analysis

Comparative moisture content, water activity (aw), and pH analyses were performed on different chocolate formulations labeled as Control, T1, T2, T3, T4, and T5, as shown in Table 2.

The moisture content, a key factor in chocolate’s stability and texture, showed no statistically significant difference (*p* ≤ 0.05) among the samples. The T1 and T2 formulations, containing premixes 1 and 2 (vitamins and minerals), exhibited moisture values comparable to the Control, indicating that adding these premixes did not substantially affect the moisture content. This confirms that incorporating vitamins and minerals into cocoa-based products does not adversely impact the moisture properties of the chocolate.

The T3, T4, and T5 formulations, which included oleogel based on Brazil nut oil and reduced cocoa butter, exhibited slightly higher moisture content compared to the Control, but this difference was not statistically significant (*p* ≥ 0.05). The increase in moisture is linked to the presence of oleogel, which forms a gel-like structure that physically retains water. The reduction in cocoa butter, an essential fat in chocolate, likely impacted the product’s texture and water retention capacity, contributing to the higher moisture content. Research has shown that modifications in fat composition can influence the moisture properties of chocolate [28].

Water activity (aw), a key property affecting stability, texture, and quality, was thoroughly evaluated for each formulation. As shown in Table 1, the Control, T1, T2, T4, and T5 formulations had similar aw values around 0.55 ± 0.01, with no significant differences (*p* ≥ 0.05). However, the T3 formulation, which contained oleogel and reduced cocoa butter, exhibited a significantly higher water activity value of 0.60 ± 0.007 (*p* ≤ 0.05). This increase indicates that the T3 formulation had more free water available compared to the Control, likely due to the combination of oleogel and reduced cocoa butter. Oleogel, a lipid-based matrix, facilitates greater water retention, boosting water activity. The reduction in cocoa butter likely disrupted fat–water interactions, leading to increased free water in the chocolate [6,29].

The results can be explained by the chemical and physical effects of incorporating oleogel and reducing cocoa butter, and by the moisture values of this sample mentioned in Table 1. Oleogel is a system that can increase water retention capacity, creating a gelatinous structure that more effectively retains water and increases water activity. On the other hand, the reduction in cocoa butter can directly impact the interaction between fat and water in chocolate. Cocoa butter, as the primary fat source in chocolate, plays a crucial role in the product’s structure and texture. Its reduction may lead to improper phase separation, allowing water to separate from cocoa particles and increasing water activity [6,29].

The pH determination can affect the stability of chocolates. The results presented in Table 1 show a statistical variation in pH values among all formulations. pH measures the concentration of hydrogen ions in a solution, and its variation reflects the acidity or alkalinity of the medium. In chocolate, acidity plays a crucial role in defining flavor, texture, product stability, and cocoa quality. It is important to note that various factors can affect pH values, including chocolate composition, cocoa content, added ingredients, and the production process. Previous studies have shown that cocoa naturally contains acids, such as acetic acid and citric acid, influencing the product’s acidity. Cocoa roasting also plays a role in pH variation, as it can lead to the degradation of organic acids, consequently reducing acidity [29].

In the case of the T4 and T5 formulations, a significant reduction in pH values was observed compared to the Control and other formulations. This suggests that these formulations are more acidic, impacting flavor, making them less sweet. This difference may be related to the synergy between the oleogel and the premix added to these formulations, indicating the presence of acidic components in the composition that decreased pH. An example is ascorbic acid (vitamin C), present in the composition of premixes 1 and 2, which is known to reduce pH in food products. Additionally, as mentioned by Santos et al. [30], the acidity values found for Brazil nut oil were 0.54 and 0.55 (%FFA).

The addition of oleogel and premixes influenced the moisture content, water activity, and pH of the chocolate formulations. While the addition of oleogel did not significantly affect moisture and water activity, it increased the free water content and altered the pH of the chocolate. The findings highlight the technological impact of fat substitutes such as oleogel in modifying the physicochemical properties of chocolate, without substantially affecting its overall stability.

### 4.2. Color Analysis

The L*, a*, b*, whiteness index, C*, and h° measurements provide detailed information about the visual appearance of chocolate, directly influencing consumer perception of quality and acceptance.

The L* value (lightness) is a measure of brightness, ranging from 0 (black) to 100 (white) in the CIELab color space. For the analyzed chocolate formulations (Control, T1, T2, T3, T4, and T5), it was observed that the variations in L* values were relatively small and did not present statistically significant differences (*p* ≤ 0.05) among them. However, in the samples with the addition of oleogel (T3, T4, and T5), there was a slight increase in lightness, indicating that the addition of the gelling agent subtly affected the system’s turbidity, despite L* values being close to those of cocoa butter. This effect may be related to the polymorphism of the crystals in the analyzed formulations. Maintaining a consistent lightness is crucial for ensuring the attractive appearance of chocolate, as it is a desirable attribute for high-quality chocolate products.

The a* and b* values represent the coordinates that define the hue angle of color concerning the red-green and yellow-blue axes, respectively, in a CIELab color space. It was observed that the T2 and T3 formulations had higher a* values compared to the Control formulation, indicating a reddish hue. Additionally, the T4 and T5 formulations exhibited lower b* values compared to the Control and the other formulations, suggesting a decrease in the yellow-blue hue. These differences may be attributed to the formulation’s composition, aligning with the results of previous studies that examined the impact of fat substitutions in chocolate products [12].

The whiteness index assesses the difference in brightness between the samples and the white standard, which is particularly relevant in chocolate. The Control, T1, and T2 formulations exhibited a lower whiteness index than the other formulations, indicating less whiteness. This variation may be influenced by specific ingredients in the premix and the oleogel, impacting the chocolate’s brightness.

The c* value measures color intensity, representing how “vibrant” or saturated the color is. In the various chocolate formulations, it was observed that the c* values did not show statistical differences (*p* ≥ 0.05) among the samples. Thus, the color saturation remained relatively consistent, regardless of the formulation. This suggests that all formulations maintained color intensity at similar levels. Saturation is an important attribute in the visual quality analysis of chocolate, as more intense colors are generally considered more attractive to consumers [31].

The h° value indicates the hue of the color concerning an angular scale, representing the direction of the color in a color space. The results showed that the h° values varied statistically among the different formulations. The T1 and T3 formulations had lower h° values, while the Control and T2 formulations had higher h° values. This indicates that the hue angle of color in T1 and T3 may be closer to red, while the Control and T2 formulations may present more bluish or greenish hue angles. These variations may be related to ingredients in the formulations that affect the direction of the color.

### 4.3. Thermal Analysis DSC

#### 4.3.1. Melting Analysis

Differential Scanning Calorimetry (DSC) is a crucial technique for evaluating the thermal and thermophysical properties of the chocolates, enabling the study of physical transitions that occur during heating. Key parameters analyzed include the onset temperature (Tonset), peak temperature (Tpeak), end temperature (Tend), and the enthalpy of fusion. The results obtained for different chocolates (Control, T1, T2, T3, T4, and T5) revealed insights into their melting behaviors (Table 4) and crystallization characteristics (Table 5). These findings provided a deeper understanding of the thermal properties of chocolate and its stability during processing and storage.

The onset temperature (Tonset) marks the beginning of the melting process, indicating the temperature at which the first fat crystals start to melt [26]. The analysis showed variations in Tonset among the different chocolate formulations; however, these differences were not statistically significant (*p* ≥ 0.05). This suggests that all formulations shared a similar onset temperature for melting. Nevertheless, chocolates containing oleogel (T3, T4, and T5) exhibited a slightly lower Tonset compared to the Control and other formulations, which may indicate a faster melting Tonset for these samples.

The peak melting temperature (Tpeak) represents the temperature at which the maximum melting rate of fat crystals occurs. Significant statistical variations were observed in Tpeak values among the formulations. For example, chocolate T4 displayed the highest melting peak, followed by T1, T5, T3, and T2. The Control chocolate showed the lowest melting peak, indicating that less energy is required to reach this melting point. This result is consistent with previous studies that determined the melting profiles of chocolate samples close to the cocoa butter melting point using Differential Thermal Analysis [28].

Tend signifies the point (temperature) at which melting is complete. Significant variations in Tend were also observed among the formulations. For instance, formulation T4 exhibited a higher Tend, suggesting a more extended thermal transition and that this chocolate takes longer to complete the melting process. Changes in crystalline structure or the presence of impurities might contribute to this variation. In contrast, chocolates T1, T2, T5, and the Control had similar Tend values with slight variations. Afoakwa et al. (2016) [32] noted that chocolates with lower fat content (25%) melt completely at higher temperatures than those with higher fat content (30–35%).

The enthalpy of fusion quantity heat is involved in the melting process and is directly related to the quantity of solids melted [33]. Significant statistical variations (*p* ≤ 0.05) were observed in the enthalpy of fusion among some chocolate formulations. For instance, formulation T1 showed a considerably lower enthalpy of fusion than the Control, suggesting a difference in the melted solids. This may be attributed to variations in ingredient composition. Chocolate T4 displayed the highest melting enthalpy, indicating that it requires more energy for the melting process than the other tested chocolates.

#### 4.3.2. Crystallization Thermal Analysis

Regarding the crystallization of chocolate, the results in Table 5 indicate that the Control chocolate had the highest temperature for the Tonset of crystallization, whereas chocolates T1, T2, T3, T4, and T5 had lower values, suggesting an earlier Tonset of crystallization, marking the initial formation of fat crystals in the chocolate. It was observed that, in general, all formulations displayed similar Tonset values, differing from the Control and being statistically significant (*p* ≤ 0.05). This suggests that the crystallization Tonset process is comparable across the samples.

The crystallization peak temperature represents the maximum temperature at which the chocolate crystallizes. Chocolates T1, T2, T3, T4, and T5 had similar values for the crystallization peak, while the Control showed a slightly higher value, reflecting the pattern observed at the Tonset of crystallization. The presence of solid compounds affected the behavior of fat crystallization due to their ability to modify the crystalline network.

Interpreting crystallization thermal values, the formulations also showed statistically significant variations (*p* ≤ 0.05). The T4 formulation exhibited a higher Tend, indicating that the crystallization process continues at higher temperatures. The crystallization enthalpy (J·g⁻^1^) reflects the amount of energy released during the crystallization process of the chocolate and is directly proportional to the fat crystals formed. Different chocolate formulations demonstrated significant variations in crystallization enthalpy. For instance, the T3 formulation showed a lower crystallization enthalpy than the Control, suggesting that few fat crystals formed.

### 4.4. Rheological Analysis

Rheological analysis is essential to understand chocolate behavior during processing and its final quality. Among the most relevant parameters, thixotropy stands out, describing a material’s ability, after being subjected to shear stress, to return to its original viscosity once the stress is removed. This property is closely related to structural changes that occur over time under shear conditions, and its evaluation is particularly important for processes involving rapid flow variations, such as tempering and coating [17,18,19].

Thixotropy is a non-linear characteristic of viscoelastic materials, often quantified by the difference between the ascending and descending shear rate curves, and the hysteresis area, which is the difference between these curves, is commonly considered as a measure of time-dependent behavior, although it is not strictly related to thixotropy. However, it is important to emphasize that not every difference observed in the shear curves indicates thixotropy, as factors such as recovery time and stress intensity influence this measurement [18].

The Casson model was adopted to describe the rheological behavior of the chocolate formulations, given its success in characterizing non-Newtonian systems with yield stress and non-linear shear behavior. However, a pertinent question arises regarding the adjustment of the Casson parameters, as the time dependence results in two viscosity curves (ascending and descending). The adjustment should be made based on the viscosity curve obtained without the influence of recovery time, which is generally the ascending curve. This procedure is widely adopted in the literature to ensure accuracy in determining rheological parameters [34].

Table 6 presents the Casson viscosity of the Control chocolate, which was 2.88 ± 0.62 Pa·s, while the experimental formulations ranged from 2.33 to 4.92 Pa·s. Casson viscosity, one of the most widely used measurements in the food industry to characterize flow resistance, reflects the complexity of the chocolate’s structural network and is highly sensitive to the interaction between suspended solids and cocoa butter, as well as interactions between ingredients like emulsifiers and oleogel systems.

The curve fitting points are the Casson yield value at the axis intercept and Casson viscosity. The curve fitting is performed using the square root function. The flow curve plot on a linear scale shows that the model function is shaped as a curve with an intercept on the shear stress axis (Figure 1; Equation (5)). In the lower shear rate range, this model curve fits better than the Bingham model. The linear curve can also help to correlate the rheological parameters with real processing conditions, such as specific shear rates during molding and tempering [17,18,19]. This method to describe thixotropy evaluates the amount of structural failure under high-shear conditions, but there is no range available to evaluate structural recovery under truly low-shear conditions. In this test, the sample is sheared at different shear rates.

Casson viscosity measures how chocolate resists flow, directly related to its ability to be molded, melted, or processed into complex shapes, such as in tempering, molding, and coating processes. Table 6 shows that formulations T3 and T4 had the highest viscosity values, indicating that these formulations were more viscous and thicker compared to the Control, T1, and T2 formulations. The presence of oleogel in formulations T3 and T4 may have contributed to the increased viscosity, as oleogels are systems made up of oils and emulsifiers that, due to their gel-like structure, increase resistance to flow and can modify chocolate fluidity, especially at high solid concentrations.

The literature confirms that the addition of structured lipids, such as oleogels, can significantly alter chocolate’s viscosity by modifying the interactions between fats and solids (cocoa and sugar) and also the interactions between ingredients like emulsifiers. Glicerina et al. [34] investigated the impact of fat composition on the rheological behavior of chocolate and found a direct correlation between fat content and increased Casson viscosity. The higher viscosity values observed in T3 and T4 formulations likely reflect higher fat or structured lipid content, corroborating these findings.

Yield stress, which was determined for the Control chocolate and ranged from 16.50 to 43.72 Pa in different formulations, reflects the force required to initiate chocolate flow. This parameter is crucial for understanding how chocolate behaves during processing, especially in stages that require continuous flow, such as extrusion and molding. The results show no significant differences (*p* ≥ 0.05) between the Control and the T1, T2, T3, and T4 formulations, suggesting that all these formulations exhibit consistent rheological behavior and require similar shear forces to initiate flow.

However, formulation T5 showed higher yield stress, indicating that this formulation exhibits more viscous behavior and greater resistance to flow. This increase can be attributed to modifications in the solid content or the addition of other structural modifiers, such as thickening agents or higher proportions of plant-based solids.

Thixotropy is a key rheological characteristic that describes chocolate’s ability to recover its viscosity after shear stress is applied. This phenomenon is particularly important in processes that involve rapid shear variations, such as tempering and coating, where chocolate’s structure can be temporarily altered but needs to recover its consistency quickly [17,18,19].

As shown in Table 6 and Figure 1, thixotropy varied significantly between formulations (*p* ≤ 0.05), with values ranging from 2599 × 10^3^ (Pa·s^−1^) to 7008 × 10^−3^ (Pa·s^−1^). Formulations T3, T4, and T5 had higher thixotropy values, suggesting that these formulations possess a more fragile structure and are more prone to viscosity reduction under constant shear stress, a characteristic that may be advantageous in coating and enrobing processes but less desirable in other types of processing, such as high-pressure molding.

Studies like those by De Jesus Silva et al. [35] demonstrated that cocoa solid content is a crucial factor in chocolate thixotropy, as higher solid content increases resistance to viscosity changes. The presence of oleogel in the T3 and T4 formulations likely contributed to the high thixotropy values observed, as oleogel systems can structure and restructure lipid networks, which can influence recovery after shear stress.

While thixotropy is often associated with hysteresis, it is important to note that hysteresis is a measure of the difference between the ascending and descending shear rate curves, indicative of irreversibility in some structuring processes. However, thixotropy extends beyond hysteresis as it involves the recovery of viscosity after shear is removed. In fact, other time-dependent effects, such as internal stress relaxation and the reconfiguration of solid and liquid phases, may be present in systems with oleogel, influencing thixotropic behavior in complex ways [2,3,17,18,19].

The Casson model is widely employed to describe non-Newtonian systems such as chocolate, particularly those that exhibit yield stress and shear-thinning behavior. In this model, yield stress (τ_0_) represents the minimum stress required to initiate flow, while Casson viscosity (ηc) characterizes the resistance to flow at low shear rates. The shear rate (γ^·^) and flow behavior are incorporated into the model, with a specific exponent to account for the non-linear relationship between shear stress and shear rate [7,12,17,18,19,24,26,29].

The fitting of the yield stress (τ_0_) and Casson viscosity (ηc) parameters was conducted based on the experimental rheological data from the various formulations. All of the R² values obtained during the fitting process were close to or above 0.9, which indicates a high degree of correlation between the experimental data and the model. This strong fit suggests that the Casson model effectively captures the rheological behavior of chocolate across different formulations, making it a reliable tool for analyzing the flow properties of chocolate in various processing conditions [17,18,19,20,24,26].

This study provides valuable insights into the rheological and structural modifications of chocolate, with a particular focus on the influence of oleogel incorporation. The rheological analysis revealed that the formulations containing oleogel (T3, T4, and T5) exhibited distinct characteristics, with higher viscosity and thixotropy, significantly affecting their processing behavior. The addition of oleogel was particularly effective in modifying the chocolate’s consistency without compromising its core structural properties, such as texture and flow behavior. In contrast, the addition of premixes without oleogel in formulations T1 and T2 did not lead to significant changes in rheological and nutritional properties [11,12].

### 4.5. Texture

The influence of the addition of a premix and oleogel on the texture properties of the chocolates is demonstrated in Figure 2, where all samples show statistically significant differences (*p* ≤ 0.05) from each other. The Control formulation exhibits the highest value, suggesting that the addition of vitamins, minerals, and oleogel interacted synergistically with the chocolate matrix, making it softer. Formulations T1 and T2, which included a premix of vitamins and minerals, were statistically different (*p* ≤ 0.05) from the others. However, the results indicated that these chocolates had a significant difference in texture (*p* ≤ 0.05) compared to the Control chocolate, with increased softness.

Formulation T3, which incorporated oleogel and reduced the amount of cocoa butter, also showed a significantly different texture (*p* ≤ 0.05) compared to the Control. This suggests that the partial substitution of cocoa butter with oleogel had a notable impact on the chocolate’s texture. Likewise, formulations T4 and T5, which combined the vitamin and mineral premix with oleogel, exhibited distinct textural properties compared to the Control.

Regarding the force (N) required to break or snap the chocolate, the Control formulation required significantly more force than the chocolates containing oleogel. This is attributed to the higher content of saturated fatty acids in cocoa butter compared to oleogel. This result aligns with previous studies, where the incorporation of plastic fats, such as oleogel, as substitutes for cocoa butter in chocolate tended to reduce its hardness, possibly due to co-crystalline softening effects [36].

These findings suggest that the addition of the vitamin and mineral premix can affect the chocolate’s texture, potentially due to interactions between these components and the chocolate matrix. The formulations in this study resulted in a more favorable chocolate structure, with enhanced softness. In line with this, research by Konar et al. [12] highlighted how variations in chocolate microstructure are linked to the interactions between ingredients, further emphasizing the complex relationship between premix, oleogel, and the chocolate matrix in modifying texture.

### 4.6. Total Lipids, Fatty Acid Profile, and Triacylglycerols

Lipid analysis was conducted on the Control and T3 samples, where a significant change in the lipid phase was observed (Table 7). The results for total fat content were as follows: 34.89 ± 1.13% for the Control and 33.21 ± 1.60% for the T3 formulation. This reduction in total fat content can be attributed to the addition of oleogel, which, as an emulsion, has a lower lipid content compared to traditional chocolate.

Saturated fatty acids, such as palmitic acid (C16:0) and stearic acid (C18:0), play a crucial role in defining the physical characteristics of chocolate [34]. The concentration of palmitic acid in the Control chocolate (29.51 ± 0.02%) was higher than that in the T3 chocolate (24.91 ± 0.15%). This reduction can be attributed to the reformulation of T3, which included the use of oleogel, rich in polyunsaturated fatty acids, and a reduction in cocoa butter, representing an 11.65% decrease in saturated fatty acids (SFAs), as indicated in Table 7. The reduction in saturated fatty acids is significant for cardiovascular health, as high concentrations of these are associated with increased risks of heart disease [37,38].

Monounsaturated fatty acids (MUFAs), particularly oleic acid (C18:1n9c), are important for sensory properties and consumer health. The evaluation from Table 7 shows that the T3 formulation had a significantly higher concentration of oleic acid than the Control chocolate, reflecting a 13.4% increase in total MUFAs. This increase can be attributed to the oleogel, which is rich in oleic acid. Studies have indicated that the intake of monounsaturated fatty acids, such as oleic acid, may contribute to a reduced risk of cardiovascular diseases [39,40].

Polyunsaturated fatty acids (PUFAs), represented in Table 7 by linoleic acid (C18:2n6c) or omega-6, are essential for human health as they are essential fatty acids. The T3 chocolate exhibited an 83.44% higher concentration of linoleic acid than the Control chocolate, with the presence of oleogel in the T3 formulation being the primary reason for this increase. Polyunsaturated fatty acids, such as linoleic acid (omega-6), are essential in the diet due to their important role in maintaining health [40].

The oleogel used in the T3 formulation is primarily composed of vegetable oils, including Brazil nut oil, which significantly contributes to the lipid profile of the chocolate. Brazil nut oil is rich in monounsaturated fatty acids, such as oleic acid, and polyunsaturated fatty acids, such as linoleic acid. It is an excellent source of essential fatty acids, which promote cardiovascular and metabolic health benefits. The use of this oleogel in chocolate results in a reformulation that reduces saturated fatty acids and increases monounsaturated and polyunsaturated fatty acids, improving the nutritional profile of the product and aligning it with cardiovascular health promotion guidelines [39,40,41,42].

The analysis of the fatty acid profile revealed that the T3 chocolate, containing oleogel, presents a substantially different lipid profile compared to the Control chocolate. These modifications, such as the reduction in saturated fatty acids and the increase in monounsaturated and polyunsaturated fatty acids, are attributed to the product reformulation and the inclusion of oleogel. These changes have significant implications for the nutritional quality of the chocolate, aligning with cardiovascular health promotion [39].

The analysis of carbon content in triacylglycerols is essential for understanding the lipid composition of food products like chocolate. Variations in the carbon content of triacylglycerols among the samples reflect changes in the lipid composition of the analyzed samples, and these changes may be influenced by the presence of oleogel. Oleogel, as a gelled system, can significantly modify the structure and composition of lipids in foods. Specifically, Brazil nut oil, which is rich in mono- and polyunsaturated fatty acids, may interact with these lipid components when combined with oleogel, altering the crystallinity and viscosity of chocolate. The characteristics of Brazil nut oil, such as its high content of essential fatty acids (like oleic and linoleic acids), may directly impact the composition of triacylglycerols in chocolate, leading to modifications in lipid structure that affect both nutritional and rheological properties.

The results presented in Table 8 show that the Control and T3 samples exhibited significant variations compared to the results of T48, T50, T52, T56, and T58. Specifically, T48, T50, T52, T56, and T58 showed a reduction in the carbon content of triacylglycerols compared to the Control and T3 samples, suggesting modifications in the lipid composition. These changes could be attributed to the addition of oleogel, which appears to have influenced the lipid structure of these samples, possibly due to the interaction between the fatty acids from Brazil nut oil and the lipids present in the chocolate formulation.

On the other hand, the result of T54 showed an increase in both the carbon content and the content of the SOS (stearic-oleic-stearic) triacylglycerol, which could have a positive nutritional impact. This increase in carbon content might be related to changes in the fatty acid composition of the triacylglycerols, favoring the presence of more saturated fatty acids like stearic acid, which contributes to the stability of the formulation and may be beneficial for health [42].

It is important to note that these variations in the carbon content of the triacylglycerols could have direct implications on the quality, texture, and stability of chocolate, even during storage. Furthermore, the main triacylglycerols present in the chocolate remained consistent between the Control and T3 samples, namely: T50 (palmitic-oleic-palmitic), T52 (palmitic-oleic-stearic), and T54 (stearic-oleic-stearic). The values for these Control and T3 samples were 95.12% and 96.47%, respectively, aligning with the value reported by Lannes (1997) [23], namely, 96.2%. This consistency underscores the key role of POP, POS, and SOS triacylglycerols in the crystallization profile of cocoa butter, which is crucial for the texture and stability of chocolate [39].These results suggest that the addition of oleogel in the formulations may have modified the lipid composition in a way that impacts the nutritional and rheological properties of the chocolate, with possible positive effects on stability and fatty acid profile.

### 4.7. Determinations of Selenium and Zinc

The analysis of selenium (Se) and zinc (Zn) in the chocolate formulations with the addition of vitamin and mineral premixes provided relevant information about the content of these nutrients in the studied samples. Only the samples with the addition of premixes 1 and 2 were analyzed, as shown in Table 9.

The results of the selenium analysis showed concentrations below the limit of detection (LOD) in all samples. Selenium is an essential micronutrient with important biological functions, including antioxidant activity [43]. The absence of selenium in the samples may reflect the low natural content of the mineral in the raw materials used or losses that may occur during the manufacturing process. Although Brazil nut oil, used in the formulations, did not show detectable levels of selenium, it is well known that Brazil nuts are a significant source of this mineral, suggesting that the extraction process may have affected its retention in the formulations. This finding suggests the possibility of considering alternative sources of selenium or fortification strategies, should the goal be to enrich the formulations with this nutrient.

Regarding zinc, variations in concentration were observed among the analyzed samples. Zinc is an essential mineral, playing a fundamental role in metabolism and immune system regulation [43]. The observed variations can be attributed to the different raw materials used, as well as the addition of the vitamin and mineral premixes, which may have contributed differently to the zinc content in the formulations. The Control (without the addition of the premixes) showed significant levels of zinc, which may have influenced the differences observed between the samples with and without the premixes. The quantitative composition of the premixes, although not specified in this study, may have contributed to these variations, highlighting the complexity of chocolate formulation and the impact of different components on the final composition.

The addition of oleogel to the formulations showed a more pronounced effect on the physicochemical properties of the chocolate, influencing characteristics such as texture and stability. This indicates that oleogel plays a relevant role in modifying the properties of the chocolate. Although the exact composition of the oleogel was not the focus of this analysis, it is evident that it has a significant impact on the characteristics of the samples, justifying its inclusion in the formulations studied.

### 4.8. Chocolate with Oleogels

Chocolates can be manufactured in various ways, depending on regulations that differ from country to country. The formulations and processes vary greatly, as do the different varieties and blends of cocoa used. The nutritional value, flavor, and structure of each chocolate depend on multiple factors, many of which are complex and difficult to define uniformly. This study focuses on the impact of oleogel addition in chocolates, discussing different oleogel formulations and their applications in the final product.

The nutritional approach is a crucial aspect addressed in the study, including factors such as caloric reduction, removal or reduction in trans and saturated fats, the addition of beneficial fats (as explored in this research), and the replacement of fat with protein or carbohydrate-based alternatives. These modifications are particularly relevant as they aim to improve the nutritional profile of chocolate without compromising its essential characteristics. Additionally, formulations for special diets can be developed, expanding the range of nutritional options available in the market. Chocolate and its derivatives can be formulated in various ways to meet a range of nutritional needs and objectives without losing their core essential properties [44,45,46]. The analysis of the impact of oleogel in chocolate formulations offers a significant contribution to the industry, enabling the creation of products with improved nutritional profiles that cater to new dietary demands.

## 5. Conclusions

The incorporation of micronutrients and the partial replacement of the lipid phase with nutritional oleogel in chocolate represents an effective strategy to improve the nutritional profile of these products. This approach allows for the enrichment of chocolate with essential vitamins and minerals while contributing to the reduction in saturated fat content, as demonstrated by the results obtained. Rheology was once more used to obtain information about the products’ structure, and it was found that the presence of different lipids strongly influenced the texture and stability of chocolate. The modifications made to the formulations resulted in a chocolate with a more balanced nutritional profile and an improved structure, without compromising its essential properties. These findings highlight the potential of using oleogel in chocolates as an alternative to meet specific nutritional demands, such as reducing saturated fat and adding essential micronutrients. Technologically, the formulations showed relevant improvements, enabling the development of products with a stronger nutritional profile, aligned with the needs of consumers who are more conscious of their diet.

This study provides a detailed analysis of the rheological and structural properties of chocolate, highlighting the influence of oleogel incorporation in the formulations. The presence of oleogel significantly modified the viscous and thixotropic properties of the chocolate without compromising its essential structural characteristics. On the other hand, the inclusion of premixes did not result in significant changes in rheological properties.

## Figures and Tables

**Table 1:** Developed formulations.

Ingredients	Percentage %					
	C	T1	T2	T3	T4	T5
Cocoa liquor	50	50	50	53	53	53
Cocoa butter	10	10	10	7.0	7.0	7.0
Sugar	39	39	39	36	36	36
Soy lecithin	0.5	0.5	0.5	0.5	0.5	0.5
PGPR	0.3	0.2	0.1	0.3	0.2	0.1
Vanillin	0.2	0.2	0.2	0.2	0.2	0.2
Premix 1	-	0.1	-	-	0.1	-
Premix 2	-	-	0.2	-	-	0.2
Oleogel	-	-	-	3.0	3.0	3.0

Note: C—Control model system of dark chocolate; T1—Chocolate with premix 1; T2—Chocolate with premix 2; T3—Chocolate with 3% oleogel; T4—Chocolate with 3% oleogel plus premix 1; T5—Chocolate with 3% oleogel plus premix 2. Oleogel: 47% Brazil nut oil + 1.5%HPMC + water.

**Table 2:** Moisture, water activity, and pH analysis.

	Moisture	Water Activity	pH
Control	3.40 ± 0.14 ^a^	0.55 ± 0.010 ^a^	5.55 ± 0.05 ^a^
T1	3.46 ± 0.26 ^a^	0.54 ± 0.005 ^a^	5.25 ± 0.06 ^b^
T2	3.38 ± 0.05 ^a^	0.55 ± 0.003 ^a^	5.10 ± 0.07 ^c^
T3	3.65 ± 0.11 ^a^	0.60 ± 0.007 ^b^	5.34 ± 0.02 ^b^
T4	3.62 ± 0.47 ^a^	0.56 ± 0.015 ^a^	4.98 ± 0.02 ^cd^
T5	3.54 ± 0.31 ^a^	0.53 ± 0.009 ^a^	4.93 ± 0.02 ^d^

Identical letters in the same column indicate values that do not differ statistically (α = 0.05); different letters in the same column indicate values that do differ statistically (α = 0.05) (n: 3).

**Table 3:** Color analysis of the samples.

	L*	a*	b*	Whiteness Index	C*	h°
Control	28.02 ± 0.59 ^a^	8.29 ± 0.57 ^a^	5.22 ± 0.72 ^a^	27.34 ± 0.64 ^a^	9.86 ± 0.46 ^a^	35.04 ± 1.11 ^a^
T1	28.98 ± 0.84 ^a^	8.03 ± 0.18 ^a^	5.43 ± 0.72 ^a^	27.98 ± 0.77 ^a^	9.76 ± 0.56 ^a^	31.86 ± 1.05 ^b^
T2	28.24 ± 0.26 ^a^	8.44 ± 0.64 ^a^	5.59 ± 0.17 ^a^	27.49 ± 0.33 ^a^	9.76 ± 0.56 ^a^	33.12 ± 1.23 ^ab^
T3	29.01 ± 0.57 ^a^	8.54 ± 0.28 ^a^	5.48 ± 0.33 ^a^	28.64 ± 0.65 ^a^	10.44 ± 0.58 ^a^	31.87 ± 1.38 ^b^
T4	29.5 ± 0.15 ^a^	8.03 ± 0.32 ^a^	4.92 ± 0.24 ^a^	28.88 ± 0.11 ^a^	9.32 ± 0.34 ^a^	32.05 ± 0.52 ^b^
T5	29.3 ± 0.39 ^a^	8.56 ±0.74 ^a^	4.83 ± 0.54 ^a^	28.41 ±0.34 ^a^	10.14 ± 0.58 ^a^	32.47 ± 1.42 ^ab^

Identical letters in the same column indicate values that do not differ statistically (α = 0.05); different letters in the same column indicate values that do differ statistically (α = 0.05).

**Table 4:** Melting thermal analysis of the samples.

	Melting Onset Tonset (°C)	Melting Peak Tpeak (°C)	Melting End Tend (°C)	Melting Enthalpy (J·g^−1^)
Control	25.45 ±0.08 ^a^	32.64 ± 0.70 ^b^	37.9 ± 1.19 ^c^	44.16 ± 0.09 ^a^
T1	25.41 ± 0.05 ^ab^	33.66 ± 0.27 ^ab^	38.74 ± 0.12 ^bc^	38.81 ± 0.63 ^b^
T2	25.44 ± 0.00 ^ab^	33.32 ± 0.24 ^b^	38.77 ± 0.03 ^bc^	43.10 ± 0.94 ^a^
T3	25.39 ± 0.04 ^ab^	33.60 ± 0.53 ^ab^	39.15 ± 0.06 ^ab^	43.97 ± 0.88 ^a^
T4	25.37 ± 0.00 ^ab^	34.80 ± 0.73 ^a^	40.44 ± 0.55 ^a^	49.04 ± 0.36 ^a^
T5	25.31 ± 0.04 ^b^	33.64 ± 0.48 ^ab^	38.35 ± 0.52 ^bc^	42.16 ± 0.59 ^b^

Identical letters in the same column indicate values that do not differ statistically (α = 0.05); different letters in the same column indicate values that do differ statistically (α = 0.05) (n: 3).

**Table 5:** Crystallization thermal analysis of the samples.

	Crystallization Tonset (°C)	Crystallization Peak (°C)	Melting Tend (°C)	Crystallization Enthalpy (J·g^−1^)
Control	24.04 ± 0.74 ^a^	14.19 ± 0.21 ^a^	0.81 ± 0.03 ^b^	25.67 ± 0.81 ^a^
T1	23.48 ± 0.05 ^a^	13.19 ± 0.09 ^b^	0.76 ± 0.05 ^b^	23.83 ± 0.46 ^c^
T2	23.22 ± 0.16 ^a^	13.27 ± 0.00 ^b^	0.75± 0.03 ^b^	22.85 ± 0.79 ^e^
T3	23.25 ± 0.15 ^b^	13.06 ± 0.02 ^b^	0.14 ± 0.05 ^c^	21.39 ± 0.88 ^f^
T4	23.14 ± 0.05 ^b^	13.19 ± 0.07 ^b^	1.18 ± 0.21 ^a^	24.01 ± 0.95 ^b^
T5	22.85 ± 0.04 ^b^	13.21 ± 0.11 ^b^	0.62 ± 0.02 ^b^	23.34 ± 0.39 ^d^

Identical letters in the same column indicate values that do not differ statistically (α = 0.05); different letters in the same column indicate values that do differ statistically (α = 0.05) (n: 3).

**Table 6:** Rheological analysis.

	Casson Viscosity (Pa·s)	Yield Stress (Pa)	Thixotropy (Pa·s^−1^) × 10^−3^	R²
Control	2.88 ± 0.62 ^ab^	21.69 ± 1.1 ^a^	6842	0.92 ± 0.04
T1	2.36 ± 0.39 ^b^	20.40 ± 0.76 ^a^	2163	0.95 ± 0.00
T2	2.33 ± 0.87 ^b^	16.50 ± 1.57 ^a^	2435	0.97 ± 0.19
T3	4.93 ± 0.28 ^a^	20.82 ± 1.47 ^a^	7276	0.94 ± 0.00
T4	4.36 ± 1.50 ^ab^	16.43 ± 0.66 ^a^	7008	0.90 ± 0.07
T5	3.49 ± 0.16 ^ab^	43.72 ± 1.94 ^b^	7781	0.94 ± 0.02

Identical letters in the same column indicate values that do not differ statistically (α = 0.05); different letters in the same column indicate values that do differ statistically (α = 0.05) (n: 3).

**Table 7:** Fatty acid profile of Control and T3 samples (n: 3).

Fatty Acids	Control	T3
Palmitic (C16:0)	29.51 ± 0.02 ^a^	24.91 ± 0.15 ^b^
Palmitoleic (C16:1)	0.55 ± 0.02	0
Stearic (C18:0)	39.97 ± 1.91 ^a^	33.05 ± 0.15 ^b^
Oleic (C18:1n9c)	28.65 ± 3.16 ^a^	33.08 ± 0.03 ^a^
Linoleic (C18:2n6c)	0.81 ± 0.15 ^b^	4.89 ± 0.53 ^a^
Arachidic (C20:0)	1.23 ± 0.05 ^a^	1.07 ± 0.01 ^b^
Saturated Fatty Acids (SFAs)	70.20 ± 3.34 ^a^	62.03 ± 0.24 ^b^
Monounsaturated Fatty Acids (MUFAs)	28.91 ± 2.85 ^a^	33.08 ± 0.03 ^a^
Polyunsaturated Fatty Acids (PUFAs)	1.23 ± 0.18 ^a^	4.89 ± 0.28 ^b^

Identical letters in the same column indicate values that do not differ statistically (α = 0.05); different letters in the same column indicate values that do differ statistically (α = 0.05) (n: 3)**.**

**Table 8:** Triacylglycerol composition based on carbon grouping of Control and T3 samples (n: 3).

Carbon Content of Triacylglycerol (%)			
	Control	T3	
T48	2.76 ± 0.27 ^a^	1.69 ± 0.08 ^b^	
T50	19.20 ± 1.34 ^a^	14.82 ± 0.23 ^b^	POP
T52	42.62 ± 1.07 ^a^	41.23 ± 0.36 ^a^	POS
T54	33.21 ± 1.66 ^b^	40.61 ± 0.40 ^a^	SOS
T56	2.22 ± 0.18 ^a^	1.62 ± 0.13 ^b^	
T58	0.02 ± 0.03 ^a^	0.03 ± 0.03 ^a^	

T48–58—Triacylglycerol-Number of carbons; POP—palmitic-oleic-palmitic; POS—palmitic-oleic-stearic; SOS—stearic-oleic-stearic. Identical letters in the same column indicate values that do not differ statistically (α = 0.05); different letters in the same column indicate values that do differ statistically (α = 0.05) (n: 3).

**Table 9:** Selenium and zinc composition in Control, T1, T2, and T5 samples (n: 2).

	Se (%)	Zn (%)
Control	<LD	1.67 ± 0.03 ^c^
T1	<LD	1.95 ± 0.06 ^b^
T2	<LD	2.10 ± 0.11 ^b^
T5	<LD	2.42 ± 0.02 ^a^

Identical letters in the same column indicate values that do not differ statistically (α = 0.05); different letters in the same column indicate values that do differ statistically (α = 0.05) (n: 3).

## Data Availability

The original contributions presented in the study are included in the article, further inquiries can be directed to the corresponding author.
